# Molecular characterisation of the NDM-1-encoding plasmid p2189-NDM in an *Escherichia coli* ST410 clinical isolate from Ghana

**DOI:** 10.1371/journal.pone.0209623

**Published:** 2018-12-21

**Authors:** Alafate Ayibieke, Wakana Sato, Samiratu Mahazu, Isaac Prah, John Addow-Thompson, Mitsuko Ohashi, Toshihiko Suzuki, Shiroh Iwanaga, Anthony Ablordey, Ryoichi Saito

**Affiliations:** 1 Department of Molecular Microbiology, Tokyo Medical and Dental University Graduate School of Medical and Dental Sciences, Tokyo, Japan; 2 Noguchi Memorial Institute for Medical Research, University of Ghana, Accra, Ghana; 3 Department of Environmental Parasitology, Tokyo Medical and Dental University Graduate School of Medical and Dental Sciences, Tokyo, Japan; 4 Department of Bacterial Pathogenesis, Tokyo Medical and Dental University Graduate School of Medical and Dental Sciences, Tokyo, Japan; Cornell University, UNITED STATES

## Abstract

Global dissemination of New Delhi metallo-β-lactamase (NDM)-producing bacteria has become a major health threat. However, there are few reports regarding the identification and characterisation of NDM-producing bacteria from West Africa, including Ghana. An *Escherichia coli* strain with resistance to meropenem was isolated from the Tamale Teaching Hospital in Ghana. Its identification and determination of antibiotic susceptibility profile were carried out using commercial systems. The antibiotic resistance mechanism was analysed by phenotypic detection kits, PCR, and DNA sequencing. Conjugation experiments, S1 nuclease pulsed field gel electrophoresis, and Southern blotting were performed. Finally, the NDM-1-harbouring plasmid was characterised using next-generation sequencing and phylogenetic analysis. The meropenem-resistant *Escherichia coli* strain EC2189 harboured *bla*_NDM-1_ and belonged to sequence type 410. *bla*_NDM-1_ was located on the IncHI type transferrable plasmid p2189-NDM (248,807 bp long), which co-carried multiple resistance genes, such as *bla*_CTX-M-15_, *aad*A1, *aac(6')-Ib*, *sul*3, *dfr*A12, and *cml*A1. p2189-NDM phylogenetically differed from previously identified *bla*_NDM-1_-positive IncHI type plasmids. A truncated Tn*125* containing *bla*_NDM-1_ was bracketed by an IS*Sm-1*-like insertion sequence upstream and by a site-specific integrase downstream. To the best of our knowledge, we have, for the first time identified and molecularly characterised an NDM-1-producing *Enterobacteriaceae* strain in Ghana with *bla*_NDM-1_ that had a novel genetic structure. Our findings indicate a possibility of NDM-1 dissemination in Ghana and underscore the need for constant monitoring of carbapenemase-producing bacteria.

## Introduction

The carbapenemase-producing Gram-negative bacteria have become a worldwide healthcare concern due to their resistance to carbapenems, which are one of the last resort antibiotics for the treatment of infectious diseases caused by multidrug-resistant bacteria [1].

*bla*_NDM_ encodes Ambler class B New Delhi metallo-β-lactamase (NDM) that hydrolyses almost all β-lactams, including carbapenems. Since its initial discovery in the last decade, *bla*_NDM-1_ and its homologues have rapidly spread worldwide and become a major public health threat [2, 3]. In the African continent, NDM-producing bacteria had been detected mostly in northern and southern Africa [3], but to date, there have been no reports of NDM-producing bacteria, including *Enterobacteriaceae*, in Ghana.

Among *Enterobacteriaceae*, most of *bla*_NDM-1_-containing plasmids can be passed on between different species by horizontal gene transfer. Moreover, *bla*_NDM-1_ is found in plasmids with diverse replicon types, including IncHI, and is usually located on mobile genetic elements, such as transposons or insertion sequences [4–6]. Therefore, it is important to characterise *bla*_NDM_-carrying plasmids to understand the mechanism of gene acquisition and to trace their spread. In this study, for the first time, we describe p2189-NDM, a *bla*_NDM-1_-harbouring plasmid, in a clinical isolate of *Escherichia coli* from Ghana, and demonstrate molecular characteristics of p2189-NDM using whole-genome sequencing.

## Materials and methods

### Ethics approval

This study was approved by both the ethics committee of the Faculty of Medicine, Tokyo Medical and Dental University (M2017-208) and the ethics committee of Noguchi Memorial Institute for Medical Research, University of Ghana (FWA 00001824). Written informed consent was obtained from all participants of the study.

### Bacterial isolate

Carbapenem-resistant *E*. *coli* strain EC2189 was isolated from a urine sample of a patient who had been hospitalised in Tamale Teaching Hospital in Ghana in 2016. Bacterial identification was performed using MALDI Biotyper (Bruker Daltonics, Karlsruhe, Germany) and VITEK MS (bioMérieux Japan, Tokyo, Japan).

### Antimicrobial susceptibility testing

Minimal inhibitory concentrations (MICs) of antibiotics were determined using MicroScan WalkAway system (Beckman Coulter, Tokyo, Japan) and interpreted according to the Clinical and Laboratory Standards Institute guideline [7]. Quality control for the MICs was performed using the reference strains *E*. *coli* ATCC 25922 and *Klebsiella pneumoniae* ATCC 700603.

### Phenotypic and genotypic detection of antibiotic resistance genes

Production of extended spectrum β-lactamases (ESBLs) and carbapenemases were determined using a MASTDISCS ID AmpC & ESBL detection set and a carbapenemase detection set, respectively (MAST diagnostics, UK). PCR and DNA sequencing for the detection of β-lactamase-encoding genes (*bla*_TEM_, *bla*_SHV_, *bla*_CTX-M,_ *bla*_OXA-1_^-^like, *bla*_KPC_, *bla*_VIM_, *bla*_IMP_, and *bla*_NDM-1_) were performed as previously described [8, 9].

### Multilocus sequence typing (MLST)

Seven housekeeping genes (*adk*, *fum*C, *gyr*B, *icd*, *mdh*, *pur*A, and *rec*A) were amplified, sequenced, and subsequently allocated an allele number according to EnteroBase (http://enterobase.warwick.ac.uk/species/index/ecoli). Sequence type (ST) was determined according to the allele combination.

### Conjugation experiment

Conjugation experiments were performed by the agar mating method as described previously with some alterations [10]. Briefly, EC2189 was conjugated with the rifampicin-resistant recipient *E*. *coli* strain C600 at the donor-to-recipient ratio of 1:10. Transconjugants were subsequently selected on BTB agar plates supplemented with 50 μg/ml rifampicin and 1 μg/ml meropenem.

### S1-nuclease pulsed-field gel electrophoresis (S1-PFGE) and Southern blot hybridisation

Genomic DNA preparations for EC2189, C600 and transconjugant strains were made in agarose plugs and digested with S1 nuclease (Takara Bio, Shiga, Japan). The linearised plasmids and partially digested DNA were separated by using the CHEF-mapper XA system (Bio-Rad, Tokyo, Japan). After staining the PFGE gel, DNA fragments were transferred to Hybond N+ membrane (GE Healthcare, Tokyo, Japan), hybridised using DIG-labelled *bla*_NDM-1_ probe, and then the signal was detected using DIG high prime DNA labelling and a detection starter kit (Roche Diagnostics, Tokyo, Japan) according to the manufacturer’s instructions.

### Plasmid analysis

Plasmids were extracted using a NucleoBond Xtra Midi kit (Takara Bio, Shiga, Japan) by following the manufacturer’s instructions and sequenced by PacBio RSII (Pacific Biosciences of California, Menlo Park, CA). SMRT analysis v2.3 was used for the *de novo* assembly of the sequenced data. The plasmid sequences were annotated using online annotation system RAST [11].

Plasmid replicon typing was conducted by using PlasmidFinder v1.3 [12]. Acquired antibiotic resistance genes were identified using ResFinder v3.0 [13]. Insertion sequences were identified using ISfinder database (https://www-is.biotoul.fr/). p2189-NDM plasmid structure and *bla*_NDM-1_ genetic contexts were compared and visualised using EasyFig v2.1 [14]. Phylogenetic analysis was performed by using MEGA7 software, generating the maximum likelihood phylogeny with 1,000 bootstrap replicates using whole plasmid sequences [15].

### Nucleotide sequence accession numbers

The complete plasmid nucleotide sequence of p2189-NDM was deposited under GenBank accession number CP029631. The draft genome sequence of strain EC2189 was deposited in the GenBank whole-genome shotgun database under accession number CP029630.

## Results and discussion

### Strain EC2189 characteristics

*E*. *coli* strain EC2189 was resistant to 13 antibiotics, including meropenem, but was intermediate to imipenem, and remained fully susceptible to fosfomycin and cefmetazole (Table 1). EC2189 produced carbapenemases and harboured *bla*_NDM-1_. MLST analysis revealed that EC2189 was related to *E*. *coli* ST410 strains with *bla*_NDM-1_ that were isolated in several countries, including Norway, UK, Switzerland, France, USA, and Poland [16]. In most of these cases, isolation of *E*. *coli* ST410 strains with *bla*_NDM-1_ was associated with the history of patients’ travel to Southeast Asia, Eastern Europe, or North Africa. Moreover, ST410 strains with *bla*_NDM-4_ and *bla*_NDM-5_ also were identified in China and Egypt, respectively [17, 18].

**Table 1:** Antibiotic susceptibility of the donor EC2189, transconjugant TcEC2189 and recipient C600 strains.

Antibiotics	Minimal inhibitory concentration (MIC) μg/ml		
	EC 2189	TcEC2189	C600
Ampicillin	>16	>16	≤4
Ampicillin/Sulbactam	>16	>16	≤4
Cefazolin	>16	>16	≤2
Cefotaxime	>32	>32	≤1
Ceftazidime	>64	>64	≤1
Cefpodoxime Proxetil	>8	>8	≤4
Cefepime	>32	>32	≤2
Cefmetazole	16	16	≤2
Imipenem	2	2	≤1
Meropenem	8	>16	≤1
Aztreonam	>8	>8	≤2
Gentamicin	>8	>8	≤1
Amikacin	>32	>32	≤8
Ciprofloxacin	>4	≤1	≤1
Levofloxacin	8	≤1	≤1
Fosfomycin	≤4	≤4	≤4

**Fig 1:**
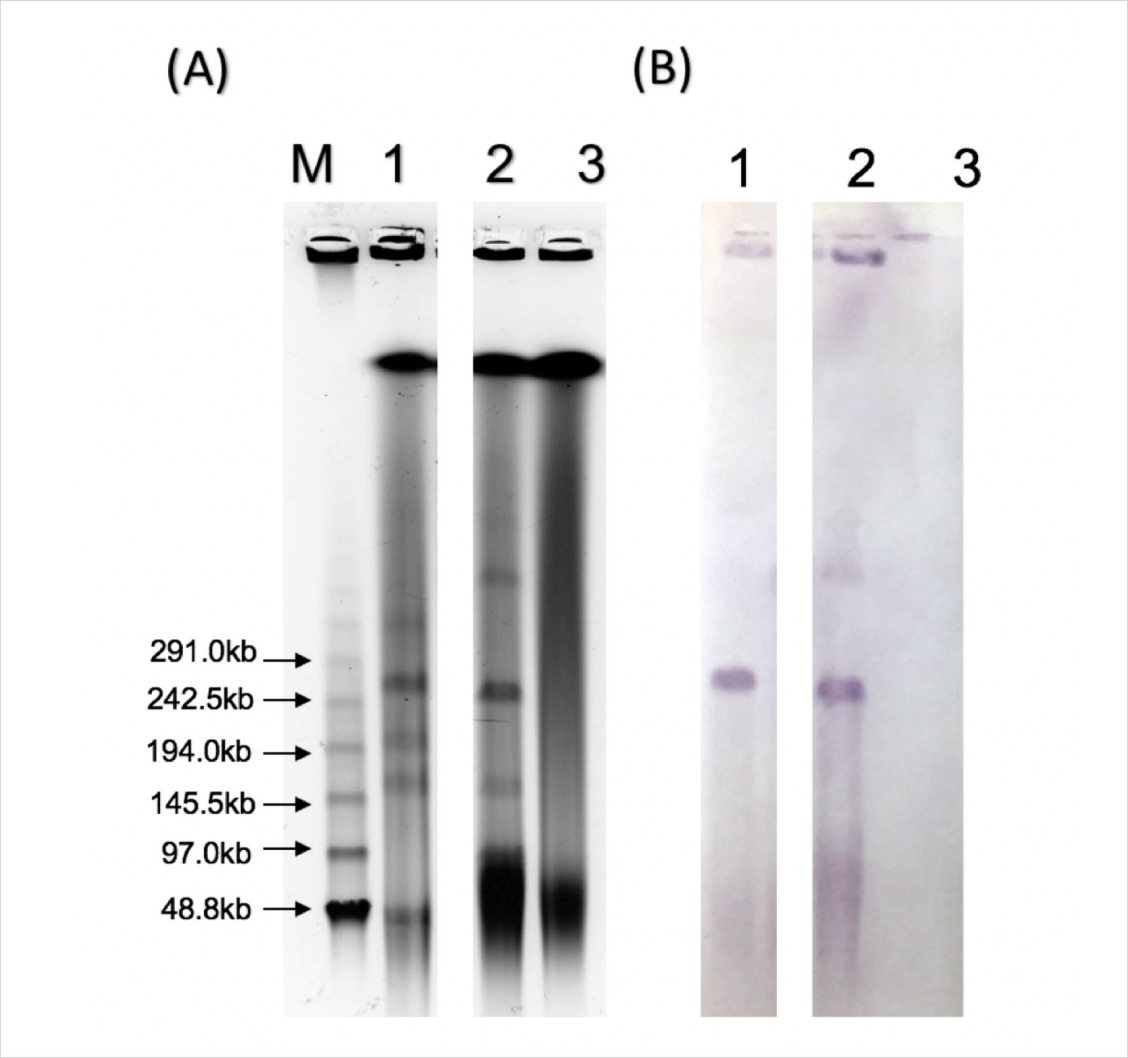
S1-nuclease pulsed-field gel electrophoresis (S1-PFGE) of the donor strain EC2189, trans-conjugant TcEC2189 and recipient C600. (A) PFGE of genomic DNA digested with S1-nuclease. (B) Southern blot hybridisation of the PFGE gel with a *bla*_NDM-1_ specific probe. Lane M: Lambda ladder; Lane 1: EC2189; Lane 2: TcEC2189; Lane 3: C600.

Meropenem-resistant transconjugant strain TcEC2189 was successfully obtained by conjugation and showed almost similar MICs to EC2189 except for fluoroquinolones (Table 1). In S1-PFGE and Southern blot analyses, *bla*_NDM-1_-positive signals were detected in both EC2189 and TcEC2189, indicating that *bla*_NDM-1_ was present on the transferrable plasmid of ~250 kb in size (Fig 1).

Furthermore, EC2189 genome was also extracted together with the plasmids and simultaneously sequenced by PacBio RSII. EC2189 possessed the S80I mutation in the quinolone resistance-determining region (QRDR) of *parC*, as well as S83L and D87N mutations in the QRDR of *gyr*A. These mutations are well-known causes of the resistance to fluoroquinolone of gram-negative bacilli, including *E*. *coli* [19, 20]. Therefore, the resistance to fluoroquinolone in EC2189 resulted from chromosome mutations in DNA gyrase and topoisomerase IV.

### Characteristics of p2189-NDM

The *bla*_NDM-1_ harbouring plasmid p2189-NDM was 248,807 bp long with the GC content of 47.8%. It encoded 287 predicted proteins (S1 Fig). p2189-NDM belonged to the IncHI1 replicon type as it possessed IncHI1A-like and IncHI1B replicons with 99.8% and 100% similarity scores to IncHI1A and IncHI1B replicons of *Salmonella* Typhi plasmid R27 (accession no. AF250878), respectively.

p2189-NDM harboured a wide range of genes that caused resistance to β-lactams (*bla*_TEM-1A,_ *bla*_CTX-M-15_, and *bla*_OXA-9_), aminoglycosides (*aad*A1, *aad*A2 and *aac(6')-Ib*), sulfonamides (*sul*3), trimethoprim (*dfr*A12), and phenicols (*cml*A1) (S1 Table). Additionally, *armA* and *bla*_OXA-1_ were only found on additional plasmids of 152,604 bp and 81,934 bp in size, respectively. As most of *bla*_NDM-1_-containing plasmids are known to carry many antibiotic resistance genes [4, 5], these results are consistent with previously published data.

**Fig 2:**
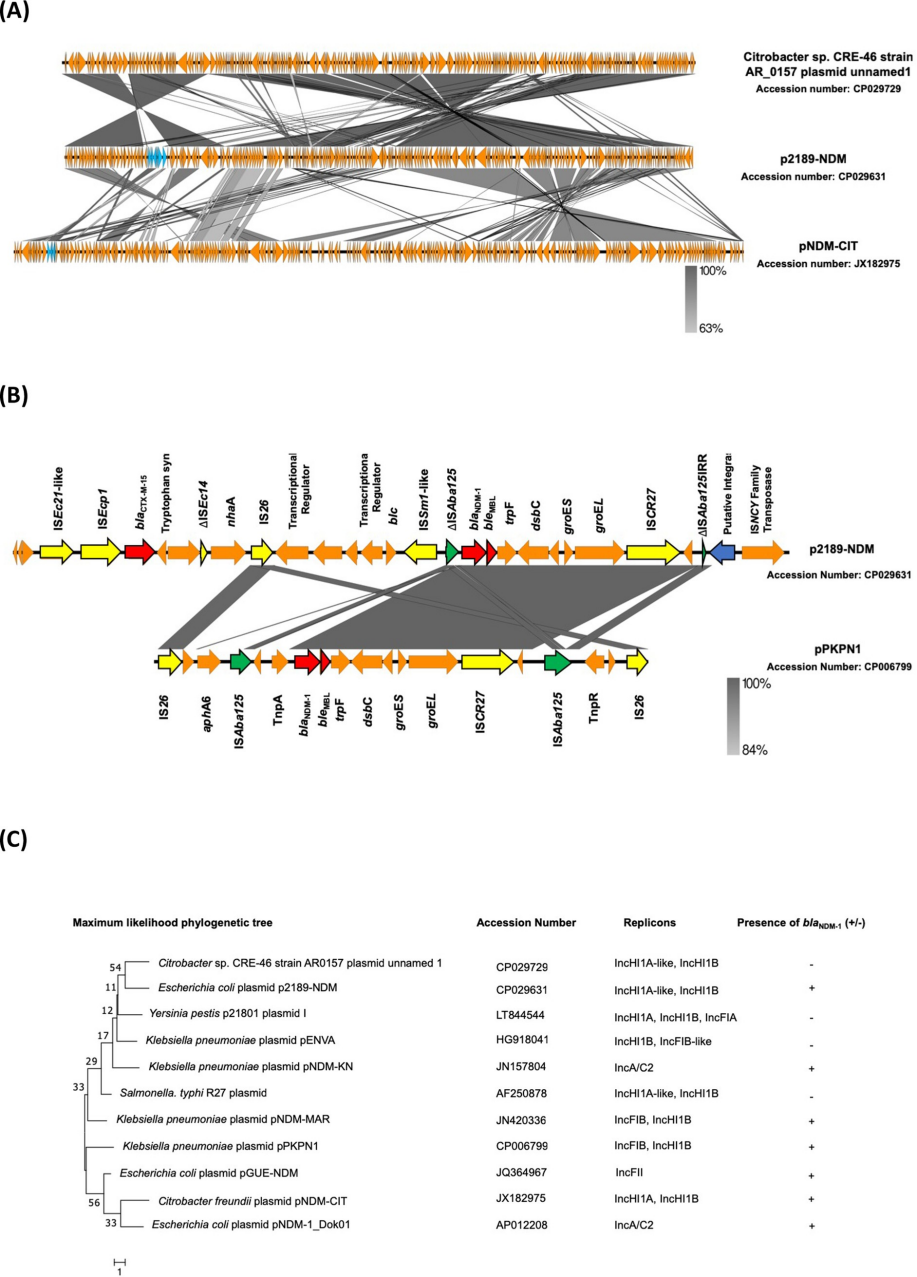
Genetic characteristics of plasmid p2189-NDM. (A) Comparisons of the plasmid backbone structure of p2189-NDM, with those of *bla*_NDM-1_-positive and negative plasmids with highest homology. The truncated Tn*125* region is highlighted in blue. Other coding sequences are coloured in orange. (B) Genetic structure comparison of the *bla*_NDM-1_-surrounding region in p2189-NDM and IncHI type plasmid pPKPN1. Antimicrobial resistance genes are coloured in red. Mobile genetic elements are coloured in yellow, with the exception of IS*125*. The insertion sequence IS*125* or truncated IS*125* are coloured in green. The site-specific integrase is coloured in blue. Other coding sequences are coloured in orange. (C) Whole genome maximum phylogenetic analysis for some *bla*_NDM-1_ positive and IncHI type *bla*_NDM-1_ negative plasmids. Phylogenetic tree was constructed using maximum likelihood method with 1,000 bootstrap replicates. Bootstrap values are shown next to branches. Plasmid accession numbers, replicon types included the plasmids and the *bla*_NDM-1_ containing information are listed next to the phylogenetic tree.

BLAST results indicated that the whole sequence of p2189-NDM had the highest homology (89% query cover and 99% identity) to the *Citrobacter* sp. CRE-46 strain AR_0157 plasmid unnamed1 (accession no. CP029729), which did not contain *bla*_NDM-1_. Among the *bla*_NDM-1_-containing plasmids, p2189-NDM showed the highest similarity to plasmid pNDM-CIT, with 60% query cover and 88% identity (accession no. JX182975) (Fig 2A). However, the gene clusters containing *bla*_NDM-1_ were inverted in p2189-NDM relative to those in pNDM-CIT. Furthermore, to investigate the genetic relationship between p2189-NDM and previously identified IncHI type plasmids with or without *bla*_NDM-1_ as well as with different types of *bla*_NDM-1_-containing plasmids, we conducted phylogenetic analysis. It revealed that p2189-NDM was genetically distinct from all other identified *bla*_NDM-1_-encoding plasmids and, as expected, was closely related to the *bla*_NDM-1_-negative *Citrobacter* sp. CRE-46 strain AR_0157 plasmid unnamed1 (Fig 2B). Collectively, these findings suggested that p2189-NDM possessed a novel backbone structure, because it was clearly separate from the previously identified *bla*_NDM-1_-encoding plasmids.

Genetic environment surrounding p2189-NDM *bla*_NDM-1_ (~25.5 kb) was compared to that of the *bla*_NDM-1_-containing IncHI type plasmid pPKPN1 of *K*. *pneumoniae* strain PittNDM01 ST14 (accession no. CP006799), with which p2189-NDM shared the highest homology among IncHI type plasmids with *bla*_NDM-1_ (Fig 2C). The genetic context of p2189-NDM was partially conserved in pPKPN1. However, the *bla*_NDM-1_-containing region in p2189-NDM was flanked by the first copy of ΔIS*Aba125* (downstream) and the second copy of ΔIS*Aba125* (upstream) and spanned ~8.3 kb, which shows truncated Tn*125*. The downstream ΔIS*Aba125* was disrupted by ISSm1-like, whereas the upstream ΔIS*Aba125* was truncated by the site-specific integrase with 100% identity to that of the *Citrobacter* sp. CRE-46 strain AR_0157 plasmid unnamed1. IS*Sm-1*-like showed 94% identity with the IS*110* family insertion sequence IS*Sm1* from *Serratia marcescens*. To the best of our knowledge, this is the first identification of IS*Sm1*-like sequence associated with the mobilisation of truncated Tn*125*. Different elements including flanking insertion sequences, singleton insertion sequences, class 1 integrons, and ISCR elements have been involved in the genetic acquisition of Tn*125*. An intact Tn*125* bounded by two copies of IS*Aba125* has often been detected in *Acinetobacter* spp. However, among *Enterobacteriaceae*, *bla*_NDM-1_ is found in the truncated ΔTn*125* region with intact or partial IS*Aba125* with the *bla*_NDM-1_ promotor sequence [4, 5, 16, 21, 22]. Therefore, our results indicated that p2189-NDM also showed a similar organization of a truncated Tn*125* surrounded by an insertion sequence. Moreover, IS*26*, which is one of the most common insertion sequences associated with the mobilisation of antibiotic resistance genes including *bla*_NDM-1_, has also been detected in p2189-NDM [4, 23]. The first copy of IS*26* was revealed between *bla*_CTX-M-15_ and *bla*_NDM-1_, and the second copy of IS*26* was found ~50 kb downstream of *bla*_NDM-1_. The IS*Ecp1*-*bla*_CTX-M-15_ module was also found upstream of *bla*_NDM-1_. Taken together, our findings suggest that p2189-NDM may have acquired *bla*_NDM-1_ from pPKPN1 or a closely related IncHI plasmid among *Enterobacteriaceae* via ΔTn*125*-mediated gene transfer, whereas the region flanked by IS26 copies had clear traces of IS-mediated homologous recombination event.

## Conclusion

In conclusion, for the first time in Ghana, we identified the NDM-1-producing *E*. *coli* strain EC2189 that harboured the transferrable plasmid p2189-NDM with multiple genes that caused resistance to antibiotics, including the *bla*_NDM-1_ gene. p2189-NDM differed phylogenetically from previously identified *bla*_NDM-1_-positive IncHI type plasmids and contained a ΔTn*125* structure known in *Enterobacteriaceae* as well as a novel IS*110* family insertion sequence, IS*Sm-1*-like. As the dissemination of *Enterobacteriaceae* possessing carbapenemases poses a significant threat to the management of infections worldwide, continuous monitoring should be strengthened to prevent the spread of carbapenemase-producing *Enterobacteriaceae* in Ghana.

## Supporting information

S1 Fig Circular map of plasmid p2189-NDM. Compared characteristics from the outside of the circle toward the centre are as follows: coding sequence on the forward strand, coding sequence on the reverse strand, GC content and GC skew. Regions with a higher GC percentage than the average one are shown by outwardly oriented light green peaks. Regions with GC percentage lower than an average is illustrated by inwardly oriented grey peaks. The height of the peak describes the difference from the average GC percentage. GC skew: the outwardly oriented light green peaks describe the region with higher G content, whereas inwardly oriented grey peaks describe the regions with higher C content. Resistance genes are shown in red arrows, and the replicons are shown in black arrows.
(PDF)

S1 Table Plasmid p2189-NDM genes confer resistance to antibiotics. (PDF)

## References

[1] Patel G, Bonomo RA. "Stormy waters ahead": global emergence of carbapenemases. Front Microbiol. 2013;4:48 Epub 2013/03/14. 10.3389/fmicb.2013.00048 23504089 PMC3596785

[2] Yong D, Toleman MA, Giske CG, Cho HS, Sundman K, Lee K, et al Characterization of a new metallo-β-lactamase gene, blaNDM-1, and a novel erythromycin esterase gene carried on a unique genetic structure in Klebsiella pneumoniae sequence type 14 from India. Antimicrobial agents and chemotherapy. 2009;53(12):5046–54. 10.1128/AAC.00774-09 19770275 PMC2786356

[3] Logan LK, Weinstein RA. The epidemiology of carbapenem-resistant Enterobacteriaceae: the impact and evolution of a global menace. The Journal of infectious diseases. 2017;215(suppl_1):S28–S36. 10.1093/infdis/jiw282 28375512 PMC5853342

[4] Wailan AM, Paterson DL, Kennedy K, Ingram PR, Bursle E, Sidjabat HE. Genomic Characteristics of NDM-Producing Enterobacteriaceae Isolates in Australia and Their blaNDM Genetic Contexts. Antimicrob Agents Chemother. 2016;60(1):136–41. Epub 2015/10/19. 10.1128/AAC.01243-15 26482302 PMC4704237

[5] Wailan AM, Sartor AL, Zowawi HM, Perry JD, Paterson DL, Sidjabat HE. Genetic Contexts of blaNDM-1 in Patients Carrying Multiple NDM-Producing Strains. Antimicrob Agents Chemother. 2015;59(12):7405–10. Epub 2015/09/21. 10.1128/AAC.01319-15 26392493 PMC4649221

[6] Doi Y, Hazen TH, Boitano M, Tsai YC, Clark TA, Korlach J, et al Whole-genome assembly of Klebsiella pneumoniae coproducing NDM-1 and OXA-232 carbapenemases using single-molecule, real-time sequencing. Antimicrob Agents Chemother. 2014;58(10):5947–53. Epub 2014/07/28. 10.1128/AAC.03180-14 25070096 PMC4187971

[7] Clinical and Laboratory Standards Institute. Performance Standards for Antimicrobial Susceptibility Testing: Twenty-fourth Informational Supplement M100-S24. Wayne: PACLSI; 2014.

[8] Dallenne C, Da Costa A, Decré D, Favier C, Arlet G. Development of a set of multiplex PCR assays for the detection of genes encoding important β-lactamases in Enterobacteriaceae. Journal of Antimicrobial Chemotherapy. 2010;65(3):490–5. 10.1093/jac/dkp498 20071363

[9] Wachino J, Yoshida H, Yamane K, Suzuki S, Matsui M, Yamagishi T, et al SMB-1, a Novel Subclass B3 Metallo-beta-Lactamase, Associated with ISCR1 and a Class 1 Integron, from a Carbapenem-Resistant Serratia marcescens Clinical Isolate. Antimicrobial Agents and Chemotherapy. 2011;55(11):5143–9. 10.1128/AAC.05045-11 WOS:000296375600027. 21876060 PMC3195065

[10] Lampkowska J, Feld L, Monaghan A, Toomey N, Schjørring S, Jacobsen B, et al A standardized conjugation protocol to asses antibiotic resistance transfer between lactococcal species. International journal of food microbiology. 2008;127(1):172–5. 18675485 10.1016/j.ijfoodmicro.2008.06.017

[11] Brettin T, Davis JJ, Disz T, Edwards RA, Gerdes S, Olsen GJ, et al RASTtk: a modular and extensible implementation of the RAST algorithm for building custom annotation pipelines and annotating batches of genomes. Sci Rep. 2015;5:8365 Epub 2015/02/10. 10.1038/srep08365 25666585 PMC4322359

[12] Carattoli A, Zankari E, García-Fernández A, Voldby Larsen M, Lund O, Villa L, et al In silico detection and typing of plasmids using PlasmidFinder and plasmid multilocus sequence typing. Antimicrob Agents Chemother. 2014;58(7):3895–903. Epub 2014/04/28. 10.1128/AAC.02412-14 24777092 PMC4068535

[13] Zankari E, Hasman H, Cosentino S, Vestergaard M, Rasmussen S, Lund O, et al Identification of acquired antimicrobial resistance genes. Journal of antimicrobial chemotherapy. 2012;67(11):2640–4. 10.1093/jac/dks261 22782487 PMC3468078

[14] Sullivan MJ, Petty NK, Beatson SA. Easyfig: a genome comparison visualizer. Bioinformatics. 2011;27(7):1009–10. Epub 2011/01/28. 10.1093/bioinformatics/btr039 21278367 PMC3065679

[15] Kumar S, Stecher G, Tamura K. MEGA7: Molecular Evolutionary Genetics Analysis Version 7.0 for Bigger Datasets. Mol Biol Evol. 2016;33(7):1870–4. Epub 2016/03/22. 10.1093/molbev/msw054 . 27004904 PMC8210823

[16] Fiett J, Baraniak A, Izdebski R, Sitkiewicz I, Żabicka D, Meler A, et al The first NDM metallo-β-lactamase-producing Enterobacteriaceae isolate in Poland: evolution of IncFII-type plasmids carrying the bla(NDM-1) gene. Antimicrob Agents Chemother. 2014;58(2):1203–7. Epub 2013/11/18. 10.1128/AAC.01197-13 24247128 PMC3910837

[17] Qin S, Zhou M, Zhang Q, Tao H, Ye Y, Chen H, et al First identification of NDM-4-producing Escherichia coli ST410 in China. Emerging microbes & infections. 2016;5(11):e118. 27876781 10.1038/emi.2016.117 PMC5148021

[18] Gamal D, Fernández-Martínez M, El-Defrawy I, Ocampo-Sosa AA, Martínez-Martínez L. First identification of NDM-5 associated with OXA-181 in Escherichia coli from Egypt. Emerging microbes & infections. 2016;5(4):e30. 27048740 10.1038/emi.2016.24 PMC4820674

[19] Ruiz J. Mechanisms of resistance to quinolones: target alterations, decreased accumulation and DNA gyrase protection. J Antimicrob Chemother. 2003;51(5):1109–17. Epub 2003/04/14. 10.1093/jac/dkg222 . 12697644

[20] Johnning A, Kristiansson E, Fick J, Weijdegård B, Larsson DG. Resistance Mutations in gyrA and parC are Common in Escherichia Communities of both Fluoroquinolone-Polluted and Uncontaminated Aquatic Environments. Front Microbiol. 2015;6:1355 Epub 2015/12/09. 10.3389/fmicb.2015.01355 26696975 PMC4673309

[21] Partridge SR, Iredell JR. Genetic contexts of blaNDM-1. Antimicrob Agents Chemother. 2012;56(11):6065–7; author reply 71. 10.1128/AAC.00117-12 23074228 PMC3486571

[22] Poirel L, Bonnin RA, Nordmann P. Analysis of the resistome of a multidrug-resistant NDM-1-producing Escherichia coli strain by high-throughput genome sequencing. Antimicrob Agents Chemother. 2011;55(9):4224–9. Epub 2011/07/11. 10.1128/AAC.00165-11 21746951 PMC3165296

[23] He S, Hickman AB, Varani AM, Siguier P, Chandler M, Dekker JP, et al Insertion Sequence IS26 Reorganizes Plasmids in Clinically Isolated Multidrug-Resistant Bacteria by Replicative Transposition. MBio. 2015;6(3):e00762 Epub 2015/06/09. 10.1128/mBio.00762-15 26060276 PMC4471558

