# Supplementary material for: Molecular characterisation of the NDM-1-encoding plasmid p2189-NDM in an Escherichia coli ST410 clinical isolate from Ghana
Source: PLoS One. 2018 Dec 21;13(12):e0209623. doi: 10.1371/journal.pone.0209623 (PMC6303030; doi:10.1371/journal.pone.0209623)

S1 Figure. Circular map of plasmid p2189-NDM.

Compared characteristics from the outside of the circle toward the centre are as follows: coding sequence on the forward strand, coding sequence on the reverse strand, GC content and GC skew. Regions with a higher GC percentage than the average one are shown by outwardly oriented light green peaks. Regions with GC percentage lower than an average is illustrated by inwardly oriented grey peaks. The height of the peak describes the difference from the average GC percentage. GC skew: the outwardly oriented light green peaks describe the region with higher G content, whereas inwardly oriented grey peaks describe the regions with higher C content. Resistance genes are shown in red arrows, and the replicons are shown in black arrows.

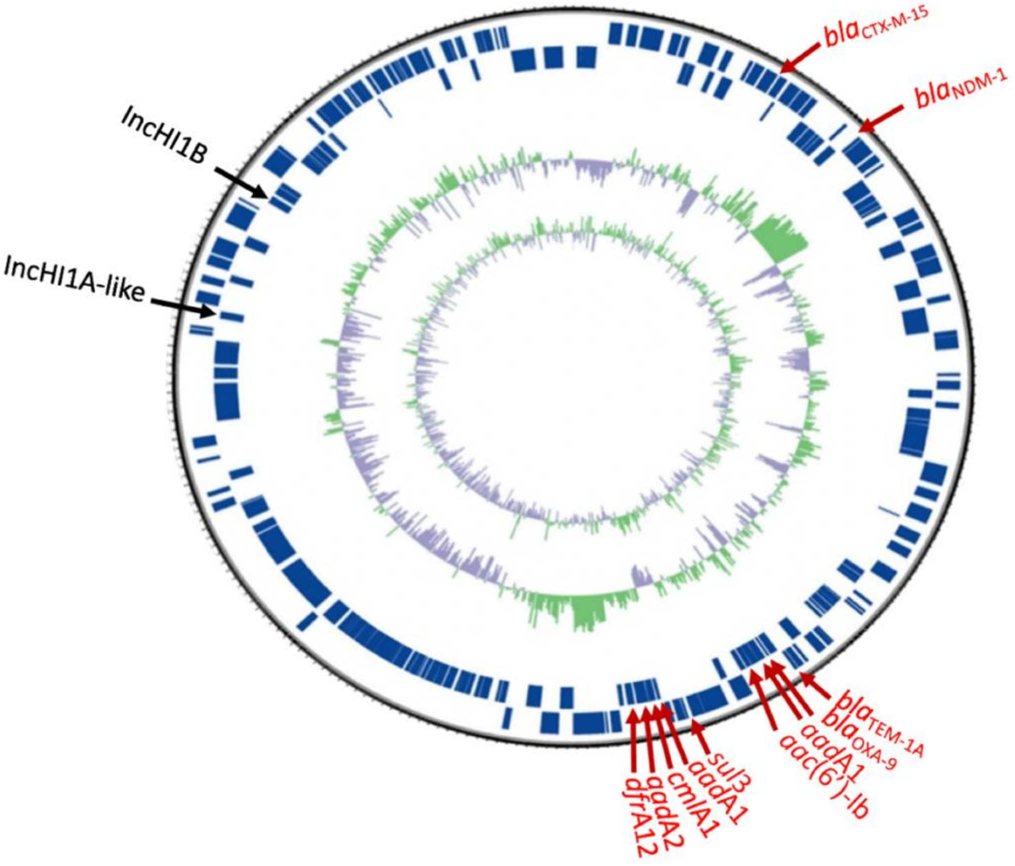

Supplement: S1 Fig — Compared characteristics from the outside of the circle toward the centre are as follows: coding sequence on the forward strand, coding sequence on the reverse strand, GC content and GC skew. Regions with a higher GC percentage than the average one are shown by outwardly oriented light green peaks. Regions with GC percentage lower than an average is illustrated by inwardly oriented grey peaks. The height of the peak describes the difference from the average GC percentage. GC skew: the outwardly oriented light green peaks describe the region with higher G content, whereas inwardly oriented grey peaks describe the regions with higher C content. Resistance genes are shown in red arrows, and the replicons are shown in black arrows. (PDF) [file pone.0209623.s001.pdf]
