# Supplementary material for: Molecular characterisation of the NDM-1-encoding plasmid p2189-NDM in an Escherichia coli ST410 clinical isolate from Ghana
Source: PLoS One. 2018 Dec 21;13(12):e0209623. doi: 10.1371/journal.pone.0209623 (PMC6303030; doi:10.1371/journal.pone.0209623)
Supplement: S1 Table — (PDF) [file pone.0209623.s002.pdf]

S1 Table. Plasmid p2189-NDM genes confer resistance to antibiotics

**Resistance genes**	**Identity (%)**	**Query/HSP**	**Position**	**Phenotype**	**Accession no**
bla$_{CTX-M-15}$	100	876/876	22366..23241	Beta-lactam resistance	DQ302097
bla$_{NDM-1}$	100	813/813	33320..34132	Beta-lactam resistance	FN396876
bla$_{TEM-1A}$	99.88	861/861	100476..101336	Beta-lactam resistance	HM749966
bla$_{OXA-9}$	100	840/840	102036..102875	Beta-lactam resistance	JF703130
*aad*A1	100	789/789	102920..103708	Aminoglycoside resistance	JQ480156
aac(6')-Ib	99.83	606/606	103778..104383	Aminoglycoside resistance	M21682
*sul*3	99.87	792/792	112677..113468	Sulphonamide resistance	AJ459418
*aad*A1	100	792/792	115150..115941	Aminoglycoside resistance	JQ414041
*cml*A1	99.84	1260/1260	116034..117293	Phenicol resistance	M64556
*aad*A2	100	780/780	117555..118334	Aminoglycoside resistance	X68227
*dfr*A12	100	498/498	118754..119251	Trimethoprim resistance	AB571791
